# Prevalence of Premenstrual Symptoms and Their Consequences on Academic Engagement in Female Students

**DOI:** 10.7759/cureus.84367

**Published:** 2025-05-18

**Authors:** Anju Chouhan, Raghuveer Choudhary, Kamla Choudhary, Islam Khan, Sajidali S Saiyad

**Affiliations:** 1 Department of Physiology, Dr. Sampurnanand Medical College, Jodhpur, IND; 2 Department of Physiology, Government Medical College, Alwar, IND; 3 Department of Physiology, Pacific Medical College and Hospital, Pacific Medical University, Udaipur, IND

**Keywords:** academic performance, behavioral impact, emotional well-being, pmdd, premenstrual syndrome, sleep disturbance, student health

## Abstract

Introduction

Premenstrual syndrome (PMS) and premenstrual dysphoric disorder (PMDD) are conditions marked by a range of emotional, behavioral, and physical symptoms that can interfere with daily functioning. Among college students, who are already navigating academic pressure and lifestyle transitions, these symptoms may significantly influence well-being and engagement.

Objective

The objective of the study is to determine the prevalence and severity of PMS and PMDD among female college students and assess their impact on academic performance and engagement.

Methods

A cross-sectional study was conducted on 227 female students (18-25 years) from Dr. S. N. Medical College, Jodhpur, and affiliated colleges. Participants completed a validated premenstrual symptom questionnaire, and academic engagement was assessed through self-reported performance metrics. Statistical analysis included chi-squared tests and regression analysis; p-values < 0.05 were considered statistically significant.

Results

While no significant association was observed between PMS/PMDD severity and academic performance (χ² = 3.307, p = 0.191), severe PMS was significantly linked to emotional (anger, anxiety, and depression), behavioral (concentration issues, hypersomnia), and social factors (impact on family, social life, and responsibilities) (p < 0.001 for all).

Conclusion

Although academic performance remained unaffected by PMS and PMDD, these conditions significantly influenced the emotional and behavioral aspects of students' lives. This underscores the importance of psychological support and awareness strategies to promote student wellness.

## Introduction

Premenstrual syndrome (PMS) and its severe form, premenstrual dysphoric disorder (PMDD), are common but often under-recognized conditions affecting women of reproductive age. PMS is characterized by a range of emotional, behavioral, and physical symptoms that occur in the luteal phase of the menstrual cycle and typically subside with the onset of menstruation [1]. PMDD, a more severe and debilitating variant, involves marked mood disturbances and significant functional impairments [2].

The prevalence of PMS among female university students varies widely across different populations, ranging from 47.8% to over 90% [1-3]. For example, a study in the United Arab Emirates highlighted a strong link between PMS and reduced academic performance [3], while another study in Ethiopia noted a 37% prevalence of PMDD, with measurable academic consequences [4]. Furthermore, a study conducted in Northern Ethiopia reported that 83.2% of participants experienced at least one PMS symptom [5].

The academic implications of PMS and PMDD are increasingly recognized. Symptoms such as irritability, fatigue, poor concentration, and emotional instability may impair cognitive functions and interfere with daily academic responsibilities [3,4]. Some reports indicate that up to 90% of female students perceive their academic performance to be negatively impacted by premenstrual symptoms, including class absenteeism, test underperformance, and reduced engagement [3]. However, findings on the statistical significance of these effects are mixed. Alhawsawi and Shiekh [6], for instance, found no significant association between PMS and academic outcomes, despite a prevalence rate of 65.4% among medical students.

Psychosocial contributors such as stress, sleep disturbances, and familial responsibilities may intensify PMS symptoms and further affect educational performance [7,8]. Despite these impacts, menstrual-related conditions often remain underdiagnosed and inadequately managed, partly due to insufficient attention from students and healthcare providers [1].

The prevalence of PMS and dysmenorrhea among medical students is high, with the majority of affected students experiencing considerable academic disruptions, including increased absenteeism. The study emphasizes the substantial impact of menstrual symptoms on academic engagement, as students frequently miss classes and struggle to maintain focus during lectures due to physical discomfort and emotional distress [9]. Nashaat et al. highlight that both PMS and dysmenorrhea significantly impair academic performance, with students reporting increased absenteeism, reduced concentration, and difficulties in completing academic tasks. The study underscores the dual burden of physical discomfort and emotional distress faced by students, which further complicates their academic engagement [10].

Mahwish et al. found that dysmenorrhea is prevalent among medical and health sciences students, with a significant impact on their academic performance. The study emphasized that students with dysmenorrhea often face difficulties in concentrating during lectures and experience disruptions in their study routines, leading to reduced academic productivity [11]. Karmacharya et al. highlight the high prevalence of dysmenorrhea among female adolescents, noting its significant disruption to daily activities, particularly academic performance. The severity of symptoms was linked to decreased school attendance and concentration, with emotional distress, anxiety, and mood disturbances further compounding academic challenges [12]. Poudel and Koirala found that dysmenorrhea is highly prevalent among secondary school girls and significantly affects their academic performance. The study highlights that dysmenorrhea leads to absenteeism and reduced focus in class, ultimately impacting the students' ability to perform well academically [13].

In light of the limited regional data-especially in the Indian medical student population-this study aims to assess the prevalence and severity of PMS and PMDD among female students at Dr. SN Medical College, Jodhpur, and examine their effects on academic performance and concentration.

## Materials and methods

This study aimed to assess the impact of PMS and PMDD on academic performance among female college students. The study was conducted at Dr. SN Medical College, Jodhpur, and its affiliated institutions, including Government Nursing College, Government Physiotherapy College, and Government Paramedical College. Ethical approval was obtained from the Institutional Ethics Committee prior to study initiation, ensuring compliance with ethical research standards. Participants were recruited voluntarily and provided informed consent before inclusion in the study.

Study design and participants

In this cross-sectional observational study, we included those female students who were aged 18-25 years, with regular menstrual cycles in the last six months, willing to participate voluntarily with written consent, and enrolled in undergraduate and graduate programs of these institutions. Female students who had a history of psychiatric disorders (e.g., major depressive disorder and anxiety disorders), were currently on hormonal therapy (oral contraceptives, hormonal replacement therapy, etc.), or used medications that could influence menstrual cycles and were unwilling to complete the questionnaire were excluded from the current study.

Sample size calculation

The sample size was calculated using the formula for single-sample proportion estimation:

$N = \left( \frac{(Z_{1-\alpha})^2 P(100-P)}{E^2} \right)$

where Z_1-α_ is the standard normal deviation for a 95% confidence interval (1.96), P is the expected prevalence of PMS/PMDD among college students (30%, based on previous studies by Jain et al. [14]), and E is the relative allowable error (20% of P).

Sample Size and Sampling

Based on a 30% estimated prevalence and 20% allowable error, 225 participants were needed. Stratified random sampling was used. The final sample is 227.

Data collection and study tools

A structured, self-administered questionnaire, which was designed to comprehensively assess premenstrual symptoms (The Premenstrual Symptoms Screening Tool [15]) [7] and their influence on academic performance [8], with demographic information (age, academic year, and lifestyle factors) and menstrual history like age at menarche, cycle length and regularity, duration of menstruation, presence of dysmenorrhea, and frequency of premenstrual symptoms, was obtained from subjects. A stratified random sampling approach was used to ensure representation across various academic years and disciplines, and confidentiality was strictly maintained.

Statistical analysis

The collected data were analyzed using Microsoft Excel (Microsoft Corp., Redmond, WA, US) and SPSS 26 software (IBM Corp., Armonk, NY, US), and results obtained were presented as descriptive and inferential statistics like chi-squared tests, and regression analyses were performed. A p-value < 0.05 indicated significance.

## Results

Table 1 presents the distribution of emotional symptoms commonly associated with PMS and PMDD among the 227 female students surveyed. The symptoms analyzed include anger, anxiety, depression, and tearfulness-each categorized by severity (not at all, mild, moderate, and severe) and cross-tabulated with PMS classification (no PMS, moderate-to-severe PMS, and PMDD).

**Table 1:** Prevalence of emotional symptoms of PMS among students (n = 227) No PMS: students who did not experience any premenstrual syndrome symptoms; moderate-to-severe PMS: students who experienced noticeable PMS symptoms that affected their daily life but did not meet criteria for PMDD; PMDD: students diagnosed with premenstrual dysphoric disorder, a severe and debilitating form of PMS characterized by significant emotional and functional impairment. Premenstrual parameters: anger/anxiety/depression/tearfulness levels: not at all: no symptoms experienced; mild: symptoms were present but manageable and did not affect functioning; moderate: symptoms were stronger and somewhat interfered with daily activities or academic performance; severe: symptoms were intense and significantly impacted daily life, requiring coping mechanisms or interventions. No. of students: number of participants who reported each level of symptom severity within each PMS category; % of total population: percentage of the total study population (n = 227) that corresponds to each symptom level.

Premenstrual parameters	Categories	No PMS	Moderate-to-severe PMS	PMDD	No. of Students	% of total population
Anger level	Not at all	52	4	0	56	24.70%
	Mild	83	14	3	100	44.04%
	Moderate	20	37	2	59	26.02%
	Severe	2	3	7	12	5.29%
Anxiety level	Not at all	62	9	0	71	31.30%
	Mild	82	19	3	104	45.80%
	Moderate	13	27	7	47	20.70%
	Severe	0	3	2	5	2.20%
Depression level	Not at all	91	11	0	102	44.90%
	Mild	57	16	5	78	34.40%
	Moderate	7	29	3	39	17.20%
	Severe	2	2	4	8	3.50%
Tearfulness level	Not at all	84	16	2	102	44.90%
	Mild	51	19	2	72	31.70%
	Moderate	21	17	4	42	18.50%
	Severe	1	6	4	11	4.80%

The most commonly reported emotional symptom was mild anger, affecting 44.04% of students, followed by mild anxiety at 45.80%. While a considerable number of students reported moderate emotional symptoms-such as moderate anger (26.02%) and moderate depression (17.20%)-only a small percentage experienced severe symptoms, with severe anger (5.29%), severe anxiety (2.20%), and severe depression (3.50%). Tearfulness was also frequently observed, with 31.70% reporting mild levels and 4.80% reporting it as severe.

**Figure 1:**
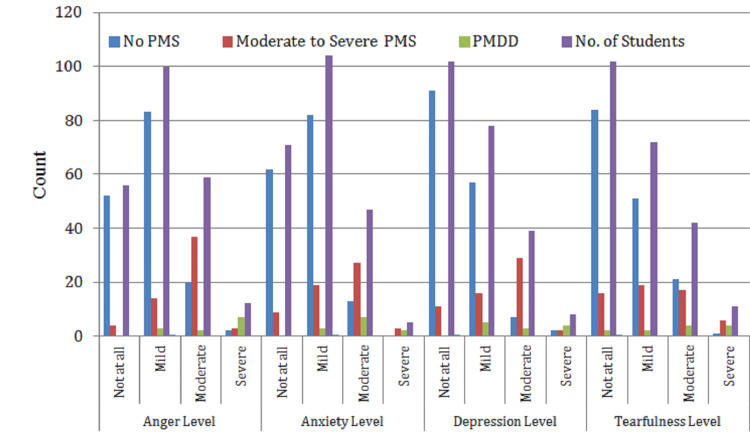
Prevalence of emotional symptoms of PMS among students (n = 227) No PMS: students who did not experience any premenstrual syndrome symptoms; moderate-to-severe PMS: students who experienced noticeable PMS symptoms that affected their daily life but did not meet the criteria for PMDD; PMDD: students diagnosed with premenstrual dysphoric disorder, a severe and debilitating form of PMS characterized by significant emotional and functional impairment. Premenstrual parameters: anger/anxiety/depression/tearfulness levels: not at all: no symptoms experienced; mild: symptoms were present but manageable and did not affect functioning; moderate: symptoms were stronger and somewhat interfered with daily activities or academic performance; severe: symptoms were intense and significantly impacted daily life, requiring coping mechanisms or interventions. No. of students: number of participants who reported each level of symptom severity within each PMS category.

Table 1 and Figure 1 highlight the high prevalence and gradation of emotional disturbances in the premenstrual phase, particularly among those with moderate-to-severe PMS and PMDD, suggesting a potential negative influence on mental well-being and daily functioning during this time.

Table 2 presents the prevalence and distribution of behavioral symptoms associated with PMS among the 227 female students surveyed. The symptoms evaluated include reduced interest in work and home activities, poor concentration, and the impact of PMS on work efficiency, relationships, family, social life, and home responsibilities. Each parameter is categorized by severity (not at all, mild, moderate, and severe) and analyzed in relation to PMS and PMDD classifications.

**Table 2:** Prevalence of behavioral symptoms of PMS among students (n = 227) No PMS: no premenstrual symptoms reported; moderate-to-severe PMS: noticeable symptoms affecting daily life, but not classified as PMDD; PMDD: severe PMS with significant emotional and functional disruption. Premenstrual parameters: low interest in work/home activities, concentration, work efficiency, relationship, family, social life, and home responsibilities: functional areas impacted by premenstrual symptoms. Symptom levels: not at all: no impact; mild: slight impact, manageable; moderate: noticeable impact, some interference; severe: strong impact, significantly disrupts daily life. No. of students: number reporting each level; % of total population: proportion of all participants (n = 227).

Premenstrual parameters	Categories	No PMS	Moderate-to-severe PMS	PMDD	No. of students	% of total population
Low interest in work	Not at all	65	2	0	67	29.5%
	Mild	67	19	3	89	39.2%
	Moderate	23	28	3	54	23.8%
	Severe	2	9	6	17	7.5%
	Total	157	58	12	227	100.0%
Low interest in home activities	Not at all	68	2	0	70	30.9%
	Mild	65	16	6	87	38.3%
	Moderate	19	32	1	52	22.9%
	Severe	5	8	5	18	7.9%
	Total	157	58	12	227	100.0%
Concentration	Not at all	62	8	1	71	31.3%
	Mild	69	21	3	93	41.0%
	Moderate	24	23	4	51	22.5%
	Severe	2	6	4	12	5.3%
	Total	157	58	12	227	100.0%
Impact on work efficiency	Not at all	80	6	1	87	38.3%
	Mild	77	37	5	119	52.4%
	Moderate	0	15	4	19	8.4%
	Severe	0	0	2	2	0.9%
	Total	157	58	12	227	100%
Impact on relationship	Not at all	94	8	0	102	44.9%
	Mild	62	27	2	91	40.1%
	Moderate	1	23	6	30	13.2%
	Severe	0	0	4	4	1.8%
	Total	157	58	12	227	100%
Impact on family	Not at all	101	18	0	119	52.4%
	Mild	54	25	3	82	36.2%
	Moderate	2	15	6	23	10.1%
	Severe	0	0	3	3	1.3%
	Total	157	58	12	227	100%
Impact on social life	Not at all	83	7	0	90	39.6%
	Mild	72	24	5	101	44.5%
	Moderate	1	27	3	31	13.7%
	Severe	1	0	4	5	2.2%
	Total	157	58	12	227	100%
Impact on home responsibilities	Not at all	91	9	0	100	44.2%
	Mild	65	21	7	93	41.2%
	Moderate	1	27	2	30	13.3%
	Severe	0	0	3	3	1.3%
	Total	157	57	12	226	100%

The data show that mild-to-moderate behavioral disruptions are highly prevalent. For instance, 39.2% of students reported mild disinterest in work, while 23.8% experienced it at a moderate level. Similarly, 41% had mild concentration issues, and 52.4% experienced a mild reduction in work efficiency, highlighting a clear pattern of functional impairment during the premenstrual phase. The impact on interpersonal domains was also notable-40.1% reported mild disruptions in relationships, and 44.5% experienced a mild effect on social life. A smaller percentage reported severe disruptions, such as 7.5% with severe disinterest in work and 1.8% experiencing severe relationship disturbances.

**Figure 2:**
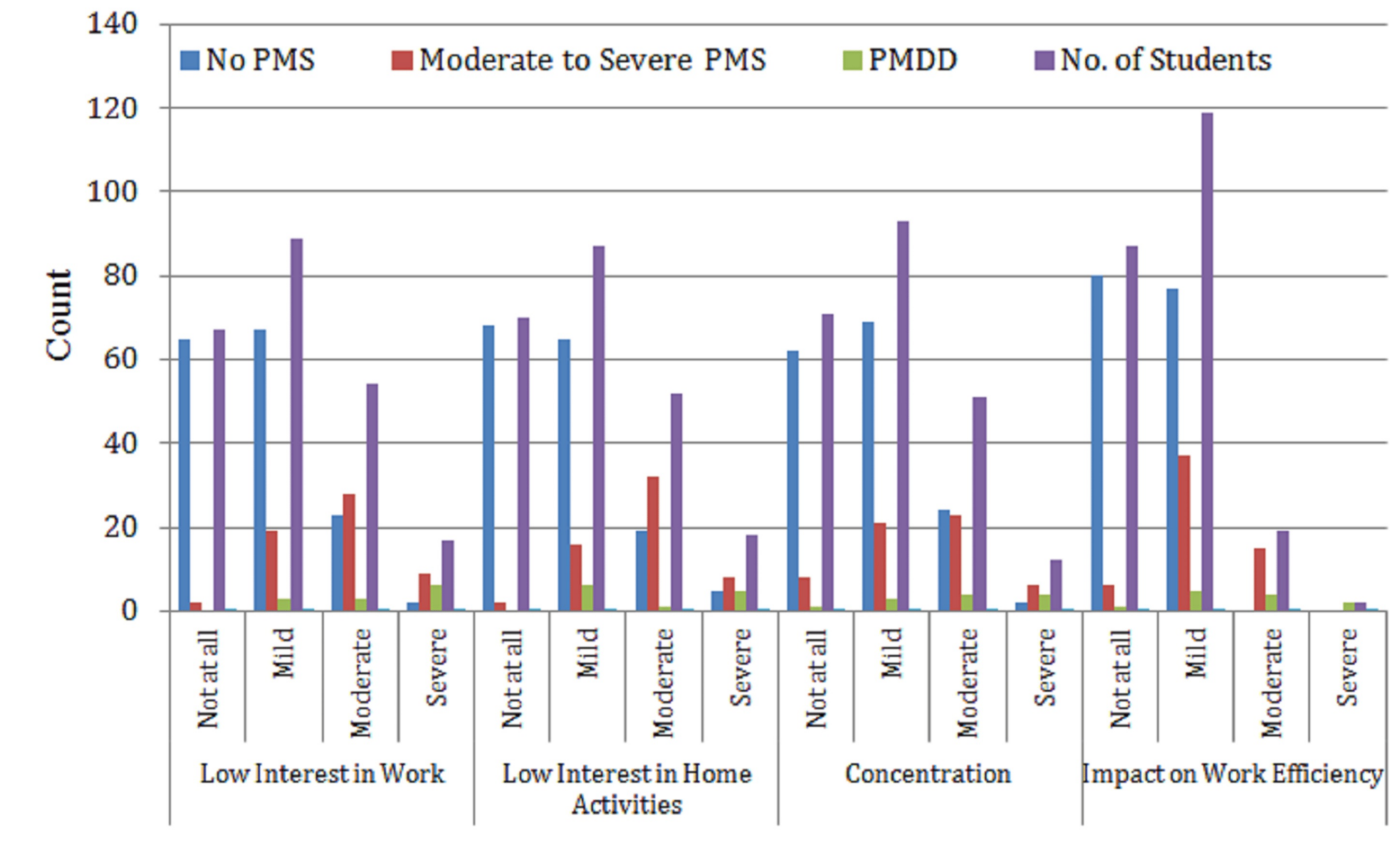
Prevalence of behavioral symptoms of PMS among students (n = 227) No PMS: no premenstrual symptoms reported; moderate-to-severe PMS: noticeable symptoms affecting daily life, but not classified as PMDD; PMDD: severe PMS with significant emotional and functional disruption. Premenstrual parameters: low interest in work/home activities, concentration, and work efficiency: functional areas impacted by premenstrual symptoms. Symptom levels: not at all: no impact; mild: slight impact, manageable; moderate: noticeable impact, some interference; severe: strong impact, significantly disrupts daily life. No. of students: number reporting each level.

**Figure 3:**
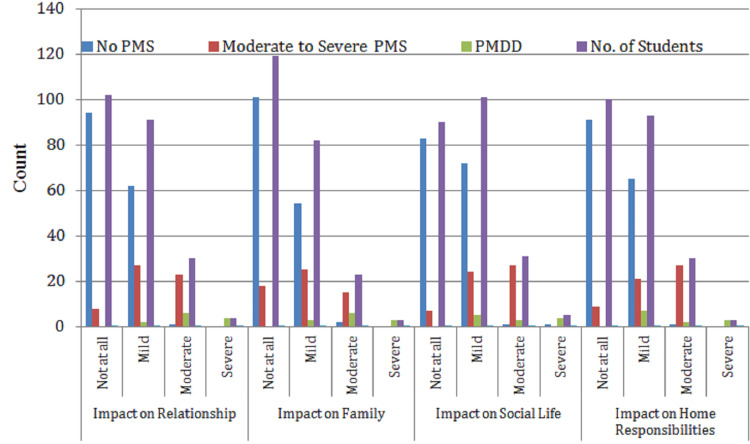
Prevalence of behavioral symptoms of PMS among students (n = 227) No PMS: no premenstrual symptoms reported; moderate-to-severe PMS: noticeable symptoms affecting daily life, but not classified as PMDD; PMDD: severe PMS with significant emotional and functional disruption. Premenstrual parameters: impact on relationship, impact on family, impact on social life, and impact on home responsibilities: functional areas impacted by premenstrual symptoms. Symptom levels: not at all: no impact; mild: slight impact, manageable; moderate: noticeable impact, some interference; severe: strong impact, significantly disrupts daily life. No. of students: number reporting each level.

Table 2 and Figures 2, 3 underscore how behavioral symptoms of PMS can extend beyond the individual to affect productivity and interpersonal dynamics, emphasizing the broader implications for academic and daily life functioning.

**Figure 4:**
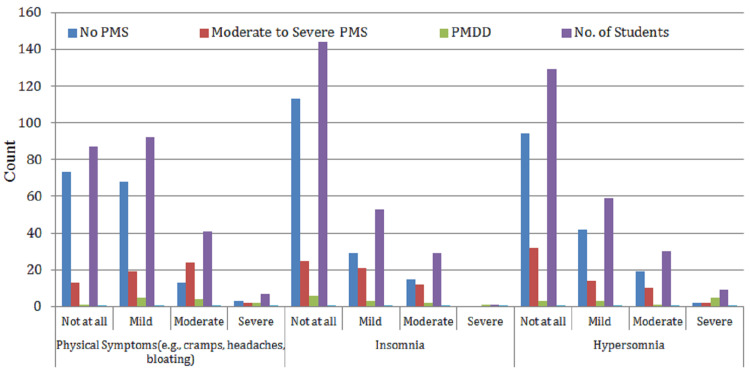
Prevalence of physical symptoms of PMS among students (n = 227) No PMS: students with no premenstrual symptoms; moderate-to-severe PMS: students with noticeable symptoms affecting daily life; PMDD: students with severe, clinically significant symptoms (premenstrual dysphoric disorder). Premenstrual parameters: physical symptoms: cramps, headaches, bloating, etc.; insomnia: difficulty falling or staying asleep; hypersomnia: excessive sleep or sleepiness. Symptom levels: not at all: no symptoms; mild: slight, manageable symptoms; moderate: noticeable symptoms, some daily impact; severe: intense symptoms, major disruption to daily life. No. of students: number of students reporting each level.

Table 3 and Figure 4 highlight the prevalence of physical symptoms associated with PMS among the 227 students included in the study. The table categorizes common physical complaints such as abdominal cramps, headaches, bloating, sleep disturbances (insomnia and hypersomnia), and their severity levels across participants with no PMS, moderate-to-severe PMS, and PMDD.

**Table 3:** Prevalence of physical symptoms of PMS among students (n = 227) No PMS: no premenstrual symptoms reported; moderate-to-severe PMS: noticeable symptoms affecting daily life, but not classified as PMDD; PMDD: severe PMS with significant emotional and functional disruption. Premenstrual parameters: low interest in work/home activities, concentration, work efficiency, relationship, family, social life, and home responsibilities: functional areas impacted by premenstrual symptoms. Symptom levels: not at all: no impact; mild: slight impact, manageable; moderate: noticeable impact, some interference; severe: strong impact, significantly disrupts daily life. No. of students: number reporting each level; % of total population: proportion of all participants (n = 227).

Premenstrual parameters	Categories	No PMS	Moderate-to-severe PMS	PMDD	No. of students	% of total population
Physical symptoms (e.g., cramps, headaches, and bloating)	Not at all	73	13	1	87	38.30%
	Mild	68	19	5	92	40.60%
	Moderate	13	24	4	41	18.10%
	Severe	3	2	2	7	3.10%
Insomnia	Not at all	113	25	6	144	63.40%
	Mild	29	21	3	53	23.30%
	Moderate	15	12	2	29	12.80%
	Severe	0	0	1	1	0.40%
Hypersomnia	Not at all	94	32	3	129	63.40%
	Mild	42	14	3	59	23.30%
	Moderate	19	10	1	30	12.80%
	Severe	2	2	5	9	0.40%

The findings show that mild-to-moderate physical symptoms are the most frequently reported, with 40.6% of students experiencing mild physical symptoms and 18.1% reporting them as moderate. Notably, 63.4% of participants did not report insomnia, but 23.3% experienced it mildly and 12.8% moderately, indicating a sleep pattern disruption in approximately one-third of the students. A similar trend was observed for hypersomnia, with 23.3% reporting mild and 12.8% reporting moderate levels of excessive sleepiness during the premenstrual phase.

While severe physical symptoms were relatively less prevalent, the overall data suggest a considerable burden of somatic discomfort among students during the premenstrual phase, which could contribute to fatigue, reduced daily functioning, and academic inefficiency. These results further emphasize the physiological dimension of PMS and its potential to interfere with students’ routine and academic performance.

Table 4 presents the statistical association between PMS/PMDD and a range of emotional, behavioral, and physical symptoms among the study population (n = 227), using the Pearson chi-squared (χ²) test. The results reveal that all analyzed symptoms-including emotional indicators like anger, anxiety, depression, and tearfulness; behavioral aspects such as low interest, impaired concentration, reduced work efficiency, and strained relationships; and physical symptoms like somatic discomfort, insomnia, and hypersomnia-showed statistically significant associations with PMS and PMDD (p < 0.001 across all variables).

**Table 4:** Association between PMS/PMDD and emotional, physical, and behavioral symptoms (n = 227) Symptom: psychological, behavioral, or physical symptom assessed in relation to PMS/PMDD; χ² (chi-squared): chi-squared test statistic indicating the strength of association between PMS severity and the symptom; df = 6, n = 227: degrees of freedom = 6; total sample size = 227; p-value: probability value indicating the statistical significance of the observed association; significance level: indicates whether the result is statistically significant; p < 0.05 is considered significant. All symptoms showed a statistically significant association with PMS/PMDD severity (p < 0.001), highlighting the broad emotional, behavioral, and social impact of premenstrual symptoms.

Symptom	χ² with df = 6, n = 227	p-value	Significance level
Anger	135.470	0.000	Significant
Anxiety	72.252	0.000	Significant
Depression	105.253	0.000	Significant
Tearfulness	46.264	0.000	Significant
Low interest	88.310	0.000	Significant
Low interest (home)	87.963	0.000	Significant
Concentration	53.193	0.000	Significant
Work efficiency	102.347	0.000	Significant
Relationships	161.395	0.000	Significant
Family	115.819	0.000	Significant
Social life	145.416	0.000	Significant
Home responsibilities	145.943	0.000	Significant
Physical symptoms	45.782	0.000	Significant
Insomnia	33.824	0.000	Significant
Hypersomnia	49.543	0.000	Significant

The strongest associations were observed in interpersonal and emotional domains, particularly relationships (χ² = 161.395), anger (χ² = 135.470), and social life (χ² = 145.416). These findings underscore the multifaceted impact of PMS and PMDD, highlighting how these conditions not only manifest physically but also significantly disrupt emotional stability, daily activities, and social functioning.

Table 4 reinforces the clinical importance of recognizing PMS and PMDD as complex conditions with broad implications for the mental health and productivity of young women, especially in academic settings.

**Figure 5:**
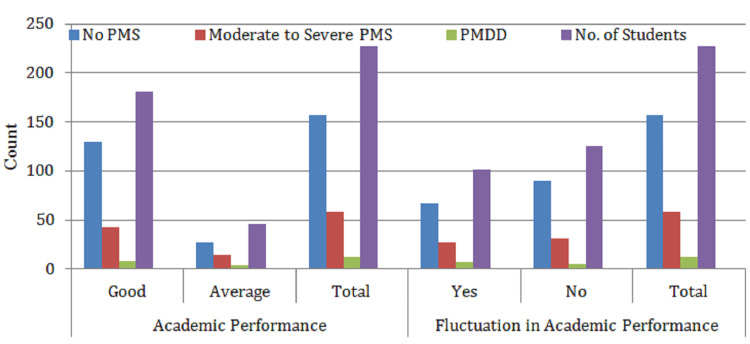
Prevalence of the effect of PMS and PMDD on academic performance and fluctuation in academic performance (n = 227) No PMS: students who did not experience any premenstrual syndrome symptoms; moderate-to-severe PMS: students with noticeable PMS symptoms affecting daily life but not qualifying as PMDD; PMDD: students diagnosed with premenstrual dysphoric disorder, a severe form of PMS. Academic parameters: academic performance: good: students reported consistently strong academic outcomes; average: students reported average or fluctuating academic performance. Fluctuation in academic performance: yes: students perceived noticeable variation in academic performance during the premenstrual period; no: students did not report any performance fluctuation. No. of students: number of respondents in each category.

Table 5 and Figure 5 illustrate the impact of PMS and PMDD on academic performance and fluctuations in academic consistency among the 227 students studied. A majority of participants (79.69%) reported maintaining good academic performance overall; however, when broken down by PMS severity, a noticeable decline is observed. Specifically, only 43 students with moderate-to-severe PMS and eight with PMDD reported good academic performance, compared to 130 students without PMS.

**Table 5:** Prevalence of effect of PMS and PMDD on academic performance and fluctuation in academic performance (n = 227) No PMS: students who did not experience any premenstrual syndrome symptoms; moderate-to-severe PMS: students with noticeable PMS symptoms affecting daily life but not qualifying as PMDD; PMDD: students diagnosed with premenstrual dysphoric disorder, a severe form of PMS. Academic parameters: academic performance: good: students reported consistently strong academic outcomes; average: students reported average or fluctuating academic performance. Fluctuation in academic performance: yes: students perceived noticeable variation in academic performance during the premenstrual period; no: students did not report any performance fluctuation. No. of students: number of respondents in each category; % of total population: percentage of the total study population (n = 227) represented in each row.

Academic parameters	Categories	No PMS	Moderate-to-severe PMS	PMDD	No. of students	% of total population
Academic performance	Good	130	43	8	181	79.69%
	Average	27	15	4	46	20.31%
	Total	157	58	12	227	100.00%
Fluctuation in academic performance	Yes	67	27	7	101	44.53%
	No	90	31	5	126	55.47%
	Total	157	58	12	227	100.00%

In terms of fluctuation in academic performance, 44.53% of students (n = 101) reported experiencing noticeable inconsistency. This fluctuation was more prominent in students with moderate-to-severe PMS (27 students) and PMDD (seven students), suggesting that premenstrual symptoms may contribute to academic instability. Despite these observed trends, further statistical analysis (see Table 6) revealed no significant association between PMS/PMDD and either academic performance or its fluctuation, indicating that while symptoms are prevalent, their direct impact on academic outcomes may be moderated by other factors.

**Table 6:** Association between PMS and PMDD on academic performance and fluctuation in academic performance χ² (chi-squared value): a statistical measure assessing the relationship between PMS severity and academic parameters; df (degrees of freedom): the number of categories minus one, used in calculating the chi-squared statistic; n = 227: total number of participants included in the analysis; p-value: probability that the observed results are due to chance. A p-value < 0.05 is typically considered statistically significant; significance level: significant: a meaningful association between PMS and the academic parameter (p < 0.05); non-significant: no statistically meaningful relationship found (p ≥ 0.05).

Academic parameters	χ² with df = 2, N = 227	p-value	Significance level
Academic performance	3.307	0.191	Non-significant
Fluctuation in academic performance	1.240	0.538	Non-significant

These findings highlight the importance of further exploring how premenstrual disorders affect students’ academic engagement, beyond just performance metrics.

Table 6 presents the statistical association between PMS/PMDD and academic performance as well as fluctuations in academic consistency among the study participants. Using the Pearson chi-squared test with a degree of freedom (df) = 2, the results indicated no statistically significant relationship between the presence or severity of PMS/PMDD and either overall academic performance (χ² = 3.307, p = 0.191) or fluctuation in academic performance (χ² = 1.240, p = 0.538).

These findings suggest that while many students with PMS or PMDD report academic challenges, the statistical analysis did not confirm a significant direct impact of PMS or PMDD on academic metrics within this sample. This implies that other mediating factors-such as coping mechanisms, support systems, or individual resilience-may influence academic outcomes despite the presence of menstrual symptoms.

## Discussion

This study highlights a notable prevalence of emotional symptoms among students experiencing PMS and PMDD. Mild anger (44.04%) and mild anxiety (45.80%) were the most commonly reported symptoms, while severe forms of anger, anxiety, depression, and tearfulness were predominantly observed in students with PMDD. Emotional symptoms like depression and tearfulness also showed an upward trend with PMS severity. These findings suggest a strong link between PMS severity and emotional instability, indicating a need for early psychological screening and support interventions among affected students.

Behavioral symptoms of PMS were found to be highly prevalent, with mild-to-moderate levels of low interest in work (39.2%), poor concentration (41%), and reduced work efficiency (52.4%) reported among a significant portion of students. PMDD cases showed higher severity, especially in symptoms like low interest in home activities and difficulty managing home responsibilities. Social and interpersonal aspects, including relationships and family interactions, were also affected. These findings underscore the significant behavioral disruptions PMS and PMDD can cause, potentially interfering with daily functioning and interpersonal relationships.

Physical symptoms were commonly reported among students, with the most prevalent being mild-to-moderate issues such as cramps, headaches, and bloating (40.6% and 18.1%, respectively). Sleep disturbances were also evident, with 23.3% experiencing mild insomnia and a similar percentage (23.3%) reporting mild hypersomnia. Although severe symptoms were less frequent, they were more pronounced among those with PMDD. These results highlight the substantial physical burden PMS can impose, which may further contribute to emotional and behavioral distress during the premenstrual phase.

Table 4 demonstrates a statistically significant association between PMS/PMDD and a wide range of emotional, physical, and behavioral symptoms. The Pearson chi-squared tests revealed significant correlations across various symptoms, including anger, anxiety, depression, and tearfulness, as well as behavioral impacts such as low interest in work and home activities, reduced concentration, and diminished work efficiency. Additionally, physical symptoms such as insomnia and hypersomnia were also significantly associated with PMS/PMDD. These findings underscore the multifaceted nature of PMS/PMDD, which not only affects emotional well-being but also disrupts daily functioning and quality of life.

Table 5 illustrates the impact of PMS and PMDD on academic performance and fluctuation in academic performance among students. The majority of students (79.69%) reported maintaining good academic performance regardless of PMS/PMDD status, while a smaller proportion (20.31%) indicated average academic performance. However, when assessing academic fluctuation, 44.53% of students reported experiencing fluctuations in their academic performance, with a higher prevalence among those with moderate-to-severe PMS (27 students) and PMDD (seven students). Despite these observations, statistical analysis did not show significant differences in academic performance or fluctuations between the groups, suggesting that although PMS and PMDD may affect students' academic life, these effects were not statistically significant in this sample.

Table 6 presents the association between PMS/PMDD and academic performance, as well as fluctuations in academic performance. The statistical analysis, using the chi-squared test, revealed that there was no significant association between PMS/PMDD and academic performance (p = 0.191) or fluctuation in academic performance (p = 0.538). Although fluctuations in academic performance were observed in a portion of the sample, the lack of statistically significant results suggests that PMS and PMDD may not have a direct or strong effect on academic outcomes in this population. These findings imply that other factors, beyond the emotional, physical, and behavioral symptoms associated with PMS/PMDD, may contribute to academic performance and fluctuation.

The present study aimed to assess the prevalence and severity of PMS and PMDD among female college students and to determine their impact on academic engagement. Although our findings did not reveal a statistically significant association between PMS/PMDD severity and academic performance (χ² = 3.307, p = 0.191), a significant correlation was observed between severe PMS and emotional (anger, anxiety, and depression), behavioral (concentration issues, hypersomnia), and social (impact on family and social life) consequences (p < 0.001 for all), highlighting the broader psychosocial impact of premenstrual disorders.

These results align with existing literature emphasizing the widespread nature and multidimensional burden of PMS among university students. Abu Alwafa et al. [8] found that 100% of participants experienced at least one PMS symptom, with a nearly universal prevalence of physical (100%), psychological (99.7%), and behavioral (85.2%) symptoms. The most common complaints included fatigue, mood disturbances, anxiety, and difficulty concentrating-symptoms that mirror the primary domains assessed in our study. Additionally, the authors reported that all PMS symptoms were significantly associated with students’ psychosocial well-being (p < 0.01), reinforcing the view that PMS poses a substantial threat to mental health and daily functioning, even in the absence of measurable academic decline [8].

Our findings are consistent with previous research [1], which observed that despite the high prevalence of dysmenorrhea and PMS, many students neglected their symptoms, leading to significant disruptions in daily functioning, including academic responsibilities such as concentration during lectures, active participation in practical sessions, and engagement in psychomotor activities. Similarly, another study [3] reported that 90% of students perceived a negative impact of PMS on academic tasks, but psychological symptoms such as anxiety and low mood were frequently normalized and under-addressed in university environments [3].

Interestingly, while emotional and behavioral symptoms were highly prevalent and impactful in our sample, they did not correspond to significant declines in academic performance. This discrepancy may be attributable to adaptive coping strategies or academic resilience. Alhawsawi and Shiekh [6] similarly found no significant link between PMS and academic outcomes despite its high prevalence among medical students (65.4%) [6].

Furthermore, lifestyle and dietary factors may modulate PMS severity. Abu Alwafa et al. [8] observed that specific eating habits, including food preferences during menstruation, adherence to diets, and herbal tea consumption, were significantly associated with various PMS symptoms (p < 0.001 to p < 0.05) [8]. These findings support conclusions drawn by Nandakumar [7], who found that higher stress levels and decreased physical activity predicted more severe PMS symptoms [7]. Such evidence underlines the need for comprehensive wellness initiatives targeting lifestyle and stress management in academic settings.

Given the psychosocial distress caused by PMS-even in the absence of academic decline-there is a pressing need for interventions that extend beyond academic performance metrics. More sensitive measures of academic engagement, such as class participation, concentration span, and time management, should be considered. One study [4] highlighted that students’ perceived academic impairment from menstrual symptoms was linked to an increased likelihood of PMDD diagnosis, suggesting that subjective burden is as critical as objective performance indicators [4].

In light of these findings, routine screening for PMS and PMDD, integrated mental health services, and psychoeducation in academic institutions are vital. Mental health professionals are key to identifying exacerbating psychosocial factors and delivering timely, supportive care [2].

Syed and Rao [9] reported a high prevalence of PMS (68%) among female medical students in India, with significant impacts on college absenteeism and academic engagement. They also found that poor dietary habits and physical inactivity contributed to symptom severity, reinforcing the need for targeted interventions and lifestyle modifications to support student well-being and academic continuity.

Nashaat et al. found that dysmenorrhea and PMS significantly affected female medical students, with 70.7% reporting mood swings and 64.6% struggling with concentration and class attendance. These symptoms moderately impacted academic performance. The study also highlighted that stress worsened symptoms, while physical activity and effective coping strategies helped reduce PMS-related disruptions [10]. Mahwish et al. found that dysmenorrhea affected 70% of medical students, impacting concentration and class attendance, which in turn disrupted academic performance. Despite coping strategies, the need for institutional support was emphasized [11].

Karmacharya et al. highlighted that dysmenorrhea significantly affected female adolescents, leading to absenteeism and reduced academic performance. They emphasized the need for effective management strategies to mitigate its impact on students' educational outcomes [12]. Similar to our findings, Poudel and Koirala reported that dysmenorrhea was associated with negative academic outcomes such as absenteeism and lack of concentration, especially among secondary school girls [13].

Limitations

Despite the valuable insights gained, this study had several limitations that should be acknowledged.

Cross-Sectional Design

The study was cross-sectional in nature, which limits the ability to establish causal relationships between PMS/PMDD symptoms and their impact on academic performance. Longitudinal studies would be better suited to assess changes over time.

Self-Reported Data

All information was based on self-reported questionnaires, which may be subject to recall bias, social desirability bias, or subjective misinterpretation of symptoms, particularly emotional and behavioral aspects.

Lack of Clinical Diagnosis

PMS and PMDD were identified based on self-assessment tools rather than clinical diagnosis by a healthcare professional, which may have led to misclassification or overestimation/underestimation of cases.

Academic Performance Measures

The study relied on students' subjective reporting of their academic performance and fluctuations, without objective academic indicators such as grade point average (GPA), attendance records, or exam scores, which limits the accuracy of the findings.

Sample Representativeness

The study population was limited to a specific demographic group (female students from one institution or region), which may affect the generalizability of the results to broader populations.

Potential Confounding Variables

Factors such as mental health status, coping strategies, sleep quality, and support systems were not controlled for, even though they may influence both PMS symptoms and academic performance.

## Conclusions

The study concludes that a significant number of female students experience emotional, behavioral, and physical symptoms related to PMS and PMDD. While these symptoms are strongly associated with psychosocial impacts, their effect on academic performance was not found to be statistically significant. These findings underscore the importance of acknowledging and addressing the broader quality-of-life challenges associated with premenstrual disorders among students.

## Appendices

Name of participant ……………………….

Signature:…………………………………

Academic Performance Questionnaire

How would you rate your overall academic performance? (Excellent, Good, Average, Below Average, Poor)

Have you noticed fluctuations in your academic performance related to your menstrual cycle? (Yes/No)

If yes, please describe the changes you've observed:

Which academic areas are most affected by PMS/PMDD symptoms (e.g., coursework, exams, and projects)?

How do PMS/PMDD symptoms impact your study habits (e.g., concentration, motivation, and time management)?
